# Caregiver burden among family caregivers of incurable cancer patients in two eastern Mediterranean countries

**DOI:** 10.1186/s12904-021-00857-5

**Published:** 2021-10-18

**Authors:** Samy A. Alsirafy, Radfan Nagy, Amneh D. Hassan, Radwa Fawzy, Ahmad A. M. Abdelhafeez, Marahim O. Husein, Mohammed A. Almashiakhi, Saad H. Alabdullateef, Saeed A. Alghamdi, Ashraf M. Elyamany

**Affiliations:** 1 Palliative Care Unit, Hemato-Oncology Department, King Saud Medical City, Riyadh, Saudi Arabia; 2 Palliative Medicine Unit, Kasr Al-Ainy Center of Clinical Oncology and Nuclear Medicine, Kasr Al-Ainy School of Medicine, Cairo University, Cairo, Egypt; 3 Oncology Department, Kasr Al-Ainy Center of Clinical Oncology and Nuclear Medicine, Kasr Al-Ainy School of Medicine, Cairo University, Cairo, Egypt; 4 The Royal Wolverhampton NHS Trust, New Cross Hospital, Wolverhampton, UK; 5 Oncology Unit, Hemato-Oncology Department, King Saud Medical City, Riyadh, Saudi Arabia; 6 Medical Oncology Department, South Egypt Cancer Institute, Assiut University, Assiut, Egypt

**Keywords:** Family caregivers, Palliative care, Incurable cancer, Caregiver burden, Activities of daily living, Egypt, Saudi Arabia, Eastern Mediterranean

## Abstract

**Background:**

Although family caregivers (FCs) play an important role in the care provided to incurable cancer patients in our region, little is known about the burden they experience.

This study was conducted to determine the prevalence of caregiver burden (CB) among FCs of incurable cancer patients in two Eastern Mediterranean countries and to identify factors that may be associated with significant CB.

**Methods:**

The study included 218 FCs, 165 from Egypt and 53 from Saudi Arabia. The 22-item Zarit Burden Interview (ZBI-22) was used to assess caregiver burden CB. Significant CB was defined as a ZBI-22 score ≥ 21. The assistance with basic ADLs was classified into 3 levels according to FCs’ assistance with early/middle/late-loss basic ADLs. The relationship between CB and the assistance with ADLs and other factors was studied.

**Results:**

The mean (SD) ZBI-22 score among FCs was 23.4 (9.3) and the majority (128/218, 59%) had significant CB. Eighty-nine percent of FCs assisted with at least one basic ADL. Assistance with late-loss basic ADLs, best supportive care treatment plan and poorer performance status were associated with higher CB (*p* < 0.0001, =0.018 and = 0.005). However, in logistic regression analysis, only assistance with late-loss ADLs was independently associated with significant CB (OR = 3.4 [95%CI:1.2–9.7], *p* = 0.024).

**Conclusion:**

A substantial proportion of FCs of incurable cancer patients in our region experience significant CB. Family caregivers assisting with late-loss basic ADLs are at risk of significant CB and should be routinely screened for CB.

## Background

In 2020, the estimated number of cancer deaths in the WHO Eastern Mediterranean region (EMRO) exceeded 450,000 deaths [1]. The only realistic treatment for these hundreds of thousands of dying cancer patients is palliative care which is ideally initiated from the date of diagnosis of incurable cancer aiming at improving their quality of life as well as that of their families [2]. In addition to being recipients of palliative care; family caregivers (FCs) of incurable cancer patients play important roles in the care of their related patients. They usually provide a lot of important tasks: assistance with activities of daily living (ADLs), administrative tasks (e.g., case management), instrumental tasks (e.g., accompanying the cancer patient to medical appointments and housekeeping tasks), navigation tasks (e.g., finding a doctor) and social support activities (e.g., providing companionship) [3–5].

Informal caregivers of cancer survivors have many unmet needs related to comprehensive cancer care, emotional and psychological wellbeing, daily activities, relationships, information and spirituality [6–8]. The important roles performed by informal caregivers and their unmet needs are associated with significant caregiver burden (CB) [9–12]. The CB experienced by caregivers of cancer patients is associated with poorer quality of life among them [13]. This in turn is expected to have a negative impact on the care they provide to cancer patients. Therefore, it is essential to identify FCs with CB and intervene to relieve their burden.

The burden of caregiving among FCs of cancer patients is generally under-researched in the Eastern Mediterranean region. A number of studies from Iran showed that FCs of patients with different stages of cancer experience significant CB which was associated with poorer quality of life and identified some factors predicting higher burden like shorter time since cancer diagnosis and higher family distress [10, 13, 14]. One study from the southern region of Saudi Arabia reported a moderate/severe CB among 50% of caregivers of hospitalized terminally ill cancer patients [15].

The aim of this study was to determine the prevalence of CB among FCs of incurable cancer patients in two Eastern Mediterranean countries (Egypt and Saudi Arabia) and to study factors that may be associated with significant CB, including FCs’ assistance with ADLs of patients.

## Methods

This observational cross-sectional study was conducted in the period from November 2019 to January 2021 and included 244 FCs of patients with incurable cancer at Kasr Al-Ainy Center of Clinical Oncology and Nuclear Medicine, Kasr Al-Ainy School of Medicine, Cairo University, Cairo, Egypt and the Hemato-Oncology Department, King Saud Medical City, Riyadh, Saudi Arabia.

### Participants

Participants were Arabic-speaking adult (> 18 years) informal FCs of adult patients with incurable cancer. Incurable cancer patients were defined as patients with advanced cancer for which no further anti-cancer treatment is possible (best supportive care plan) / anti-cancer treatment is given with palliative intent (palliative anti-cancer treatment plan). Palliative care is ideally initiated for these patients from the date of diagnosis of incurable cancer and sometimes it is the only possible management plan. Only one FC per patient was included and non-family informal caregivers were excluded.

Purposive-convenience sampling method was used to recruit FCs accompanying their related patients attending the oncology outpatient clinic or those admitted to the oncology inpatient ward. Family caregivers were approached to participate in the study and interviewed by authors RN, RF and AA in Egypt and by AH and MOH in Saudi Arabia.

### Measurement tools and data collection

Caregiving burden among FCs was assessed using the 22-item Zarit Burden Interview (ZBI-22). We used the Arabic for Saudi Arabia version of the ZBI-22 [16] which was obtained from Mapi Research Trust, Lyon, France (https://eprovide.mapi-trust.org/). Each of the ZBI-22 items is scored on a 5-point scale ranging from 0 to 4. The total score is the sum of the 22 items score and ranges from 0 to 88 with higher scores indicating higher burden. Missing interview items were replaced by the mean score of answered items. The sum of interviews with replaced missing values was rounded to the nearest integer. Interviews with > 6 missing items were excluded from analysis [17]. Participants with a ZBI score ≥ 21 were considered to have significant burden [18].

The assistance provided by FCs with the following basic ADLs was assessed: dressing, combing hair, brushing teeth, bathing, toilet use, cleansing, moving between locations, transfer to/from chair, bed mobility and eating. The level of ADLs loss was graded according to Morris et al. [19] into early loss (dressing and personal hygiene), middle loss (toilet use, transfer and locomotion) and late loss (bed mobility and eating). Bathing was considered middle loss ADLs. The level of assistance provided by FCs with ADLs was graded as level 1 (no assistance / assistance with any early loss ADL), level 2 (assistance with any middle loss ADL ± assistance with any early loss ADL) and level 3 (assistance with any late loss ADL ± assistance with any early/middle loss ADL).

In addition to caregiver’s burden and assistance with ADLs; information about characteristics of FCs, patients and cancer was collected (Table 1).

**Table 1:** Characteristics of 218 family caregiver and patient dyads

Variable	Description	
***Family caregivers***		
**Age** (*n* = 215)	Median (IQR)	35 (29–44)
**Sex** (*n* = 218)		
Female	*n* (%)	127 (58.3)
Male	*n* (%)	91 (41.7)
**Marital status** (*n* = 217)		
Married	*n* (%)	149 (68.7)
Single	*n* (%)	53 (24.4)
Divorced	*n* (%)	11 (5.1)
Widow	*n* (%)	4 (1.8)
**Education** (*n* = 218)		
University	*n* (%)	91 (41.7)
High school	*n* (%)	66 (30.3)
Less than high school	*n* (%)	41 (18.8)
Illiterate	*n* (%)	20 (9.2)
**Employment** (*n* = 218)		
Full time	*n* (%)	61 (28)
Part time	*n* (%)	28 (12.8)
Stopped for caregiving	*n* (%)	31 (14.2)
None	*n* (%)	98 (45)
**Relation to the patient** (*n* = 218)		
Daughter	*n* (%)	60 (27.5)
Son	*n* (%)	53 (24.3)
Sister	*n* (%)	29 (13.3)
Husband	*n* (%)	22 (10.1)
Wife	*n* (%)	14 (6.4)
Brother	*n* (%)	12 (5.5)
Other	*n* (%)	28 (12.8)
**Comorbidities** (*n* = 204)		
Back pain	*n* (%)	27 (13.2)
Hypertension	*n* (%)	20 (9.8)
Diabetes mellitus	*n* (%)	10 (4.9)
Other	*n* (%)	21 (10.3)
**Living with the patient** (*n* = 218)	*n* (%)	153 (70.2)
**Number of other caregivers** (*n* = 209)	Median (IQR)	2 (1–3)
***Patient characteristics***		
**Age** (*n* = 217)	Median (IQR)	55 (46–63)
**Sex** (*n* = 218)		
Female	*n* (%)	164 (75.2)
Male	*n* (%)	54 (24.8)
**1ry cancer** (*n* = 214)		
Breast	*n* (%)	77 (36)
Colorectal	*n* (%)	31 (14.5)
Lung	*n* (%)	28 (13.1)
Pancreas	*n* (%)	17 (7.9)
Hematological	*n* (%)	12 (5.6)
Sarcoma	*n* (%)	10 (4.7)
Other	*n* (%)	39 (18.2)
**Distant metastases** (*n* = 197)	*n* (%)	168 (85.3)
**Aware of cancer diagnosis** (*n* = 218)	*n* (%)	186 (85.3)
**Expecting cure for his/her cancer** (*n* = 185)	*n* (%)	140 (75.7)
**Treatment plan** (*n* = 214)		
Best supportive care	*n* (%)	49 (22.9)
Palliative anti-cancer treatment	*n* (%)	165 (77.1)

### Sample size

Using the 10 outcome events per predictor variable (EPV) rule and considering a 70% prevalence of CB using the ZBI-22 cutoff score of 21 [20–24], at least 214 participants were needed for logistic regression analysis including 15 predictor variables. Anticipating a 10% exclusion rate, the sample size was further increased to 236 participants.

### Statistical methods

Categorical data were described as numbers and percentages while continuous as means and standard deviations. Independent sample t-test was used to determine the significance of difference in continuous variables between two groups and one way ANOVA for more than two groups. Pearson’s correlation coefficient was used to study the significance of correlation between two continuous variables.

Statistical analysis was done using MedCalc® Statistical Software version 20 (MedCalc Software Ltd., Ostend, Belgium; https://www.medcalc.org; 2021).

## Results

From 244 participating FCs, 218 (89.3%) were included in the analysis, 23 (9.4%) were excluded and 3 (1.2%) withdrew from the study. The causes of exclusion were missing patient’s data (11), repeated patient (5), > 6 missing ZBI items (2), no incurable cancer (2), no confirmed cancer diagnosis (1), < 18 years of age (1) and being a friend (1). 165 (75.7%) FCs were recruited from Egypt and 53 (24.3%) from Saudi Arabia. 166 (54.2%) FCs were interviewed in the outpatient setting and 98 (45.8%) in the inpatient.

The characteristics of included FCs, patients and cancer are illustrated in Table 1. As perceived by FCs, the majority of patients were aware of the diagnosis of cancer but not its prognosis. The majority of diagnosis-aware patients expected cure for their disease.

The details of assistance with ADLs provided by FCs and the level of assistance are shown in Table 2. The majority (194/218, 89%) of FCs assisted with at least one of the screened ADLs and three-quarters of them provided level 3 assistance with basic ADLs.

**Table 2:** Family caregivers’ assistance with basic activities of daily living and its level

		***n*** (%)
**Assistance with early-loss ADL**		
Dressing (*n* = 217)		146 (67.3)
Combing hair (*n* = 217)		102 (47)
Brushing of teeth (*n* = 213)		68 (31.9)
Any early loss ADL (*n* = 218)		153 (70.2)
**Assistance with middle-loss ADL**		
Toilet use (*n* = 217)		149 (68.7)
Cleansing (*n* = 215)		84 (39.1)
Moving between locations (*n* = 216)		175 (81)
Transfer to/from chair (*n* = 216)		155 (71.8)
Bathing (*n* = 216)		122 (56.5)
Any middle loss ADL (*n* = 218)		190 (87.2)
**Assistance with late-loss ADL**		
Bed mobility (*n* = 216)		149 (69)
Eating (*n* = 216)		136 (63)
Any late loss ADL (*n* = 217)		167 (77)
**Level of assistance provided by family caregiver with basic ADLs** (*n* = 218)		
Level 1 (no assistance / assistance with early-loss ADL)		26 (11.9)
Level 2 (assistance with middle-loss ADL ± assistance with early-loss ADL)		25 (11.5)
Level 3 (assistance with late loss ADL ± assistance with early / middle-loss ADL)		167 (76.6)

The majority (209/218, 96%) of participating FCs answered all items of the ZBI-22. The mean (SD) ZBI-22 score was 23.4 (9.3) and the median (IQR) was 23 (16–31). Using the ZBI-22 cutoff score of 21, 128/218 (58.7%) FCs had significant CB (total score ≥ 21). The details of the response of FCs to the 22 items of the ZBI-22 are shown in Table 3.

**Table 3:** Response of family caregivers to the ZBI-22 items

Item	*n.*	Response *n.* (%)					Mean (SD)
		0 (never)	1 (rarely)	2 (sometimes)	3 (quite frequently)	4 (nearly always)	
1. Do you feel that your relative asks for more help than he/she needs?	218	133 (61)	22 (10.1)	35 (16.1)	24 (11)	4 (1.8)	0.83 (1.2)
2. Do you feel that because of the time you spend with your relative that you don’t have enough time for yourself?	218	117 (53.7)	26 (11.9)	49 (22.5)	23 (10.6)	3 (1.4)	0.94 (1.2)
3. Do you feel stressed between caring for your relative and trying to meet other responsibilities for your family or work?	217	86 (39.6)	23 (10.6)	59 (27.2)	41 (18.9)	8 (3.7)	1.36 (1.3)
4. Do you feel embarrassed over your relative’s behaviour?	218	184 (84.4)	15 (6.9)	15 (6.9)	3 (1.4)	1 (0.5)	0.27 (0.7)
5. Do you feel angry when you are around your relative?	217	194 (89.4)	14 (6.5)	8 (3.7)	1 (0.5)	0	0.15 (0.5)
6. Do you feel that your relative currently affects your relationship with other family members or friends in a negative way?	217	162 (74.7)	19 (8.8)	30 (13.8)	6 (2.8)	0	0.45 (0.8)
7. Are you afraid what the future holds for your relative?	216	35 (16.2)	7 (3.2)	46 (21.3)	101 (46.8)	27 (12.5)	2.36 (1.2)
8. Do you feel your relative is dependent upon you?	217	15 (6.9)	16 (7.4)	46 (21.2)	94 (43.3)	46 (21.2)	2.65 (1.1)
9. Do you feel strained when you are around your relative?	217	156 (71.9)	21 (9.7)	31 (14.3)	7 (3.2)	2 (0.9)	0.52 (0.9)
10. Do you feel your health has suffered because of your involvement with your relative?	217	150 (69.1)	26 (12)	29 (13.4)	12 (5.5)	0	0.55 (0.9)
11. Do you feel that you don’t have as much privacy as you would like, because of your relative?	217	151 (69.6)	29 (13.4)	25 (11.5)	11 (5.1)	1 (0.5)	0.54 (0.9)
12. Do you feel that your social life has suffered because you are caring for your relative?	217	132 (60.8)	23 (10.6)	47 (21.7)	13 (6)	2 (0.9)	0.76 (1.1)
13. Do you feel uncomfortable about having friends over, because of your relative?	216	167 (77.3)	21 (9.7)	18 (8.3)	9 (4.2)	1 (0.5)	0.41 (0.9)
14. Do you feel that your relative seems to expect you to take care of him/her, as if you were the only one he/she could depend on	215	37 (17.2)	12 (5.6)	42 (19.5)	89 (41.4)	35 (16.3)	2.34 (1.3)
15. Do you feel that you don’t have enough money to care for your relative, in addition to the rest of your expenses?	218	43 (19.7)	15 (6.9)	59 (27.1)	81 (37.2)	20 (9.2)	2.1 (1.3)
16. Do you feel that you will be unable to take care of your relative much longer?	217	163 (75.1)	26 (12)	25 (11.5)	3 (1.4)	0	0.39 (0.7)
17. Do you feel you have lost control of your life since your relative’s illness?	217	124 (57.1)	26 (12)	46 (21.2)	19 (8.8)	2 (0.9)	0.84 (1.1)
18. Do you wish you could just leave the care of your relative to someone else?	218	184 (84.4)	18 (8.3)	13 (6)	3 (1.4)	0	0.24 (0.6)
19. Do you feel uncertain about what to do about your relative?	216	115 (53.2)	19 (8.8)	61 (28.2)	20 (9.3)	1 (0.5)	0.95 (1.1)
20. Do you feel you should be doing more for your relative?	218	51 (23.4)	19 (8.7)	63 (28.9)	74 (33.9)	11 (5)	1.89 (1.3)
21. Do you feel you could do a better job in caring for your relative?	218	35 (16.1)	13 (6)	83 (38.1)	77 (35.3)	10 (4.6)	2.1 (1.1)
		0 (not at all)	1 (a little)	2 (moderately)	3 (quite a bit)	4 (extremely)	
22. Overall, how burdened do you feel in caring for your relative?	218	120 (55)	37 (17)	37 (17)	22 (10.1)	2 (0.9)	0.85 (1.1)

The level of assistance with basic ADLs, best supportive care treatment plan and higher ECOG score were significantly associated with CB in univariate analysis (Table 4). Logistic regression analysis showed that only level 3 assistance with basic ADLs independently associated with CB.

**Table 4:** The relationship between caregiver burden and the studied variables

		Univariate analysis			Regression analysis	
		Caregiver burden			OR (95%CI)	***P*** value
		No (ZBI-22 score < 21)	Yes (ZBI-22 score ≥ 21)			
		***n*** (%)		P value		
**Level of assistance with basic ADLs provided by caregivers**	Level 1 (no assistance / assistance with early-loss ADLs)	20 (76.9)	6 (23.1)	**< 0.0001**	Ref.	
	Level 2 (assistance with middle-loss ADL ± assistance with early-loss ADL)	14 (56)	11 (44)		1.79 (0.51–6.3)	0.367
	Level 3 (assistance with late-loss ADL ± assistance with early / middle-loss ADL)	56 (33.5)	111 (66.5)		3.38 (1.18–9.72)	**0.024**
**Sex of caregiver**	Female	54 (42.5)	73 (57.5)	0.662	–	–
	Male	36 (39.6)	55 (60.4)		–	
**Sex of patient**	Female	71 (43.3)	93 (56.7)	0.295	–	–
	Male	19 (35.2)	35 (64.8)		–	
**Marital status of caregiver**	Married	65 (43.6)	84 (56.4)	0.248	–	–
	Other	24 (35.3)	44 (64.7)		–	
**Education level of caregiver**	High school / University	63 (40.1)	94 (59.9)	0.506	–	–
	Less than high school	20 (48.8)	21 (51.2)		–	
	Illiterate	7 (35)	13 (65)		–	
**Employment of caregiver**	No	54 (41.9)	75 (58.1)	0.836	–	–
	Yes	36 (40.4)	53 (59.6)		–	
**Relation to the patient**	Daughter / son	45 (39.8)	68 (60.2)	0.263	–	–
	Sister / brother	19 (46.3)	22 (53.7)		–	
	Wife / Husband	11 (30.6)	25 (69.4)		–	
	Other	15 (53.6)	13 (46.4)		–	
**Caregiver living with the patient**	No	31 (47.7)	34 (52.3)	0.211	–	–
	Yes	59 (38.6)	94 (61.4)		–	
**Caregiver has comorbidity**	No	61 (42.7)	82 (57.3)	0.661	–	–
	Yes	24 (39.3)	37 (60.7)		–	
**Primary cancer**	Breast	37 (48.1)	40 (51.9)	0.099	–	–
	Other	50 (36.5)	87 (63.5)		–	
**Anti-cancer treatment plan**	Best supportive care	13 (26.5)	36 (73.5)	**0.018**	Ref.	
	Palliative anti-cancer	75 (45.5)	90 (54.5)		0.75 (0.34–1.67)	0.482
		**Mean (SD)**		**P value**		
**Age of caregiver**		35.8 (10.7)	37 (11)	0.399	–	–
**Age of patient**		55.2 (13.6)	53.8 (13.5)	0.446	–	–
		**Median (IQR)**		**P value**		
**Number of other carers**		2 (1–3)	2 (1–3)	0.407	–	–
**ECOG performance scale**		2 (2–3)	3 (2–3)	**0.005**	1.34 (0.88–2.04)	0.177

*ECOG* Eastern Cooperative Oncology Group, *IQR* Inter-quartile range, *OR* Odds ratio, *SD* Standard deviation

**Fig. 1:**
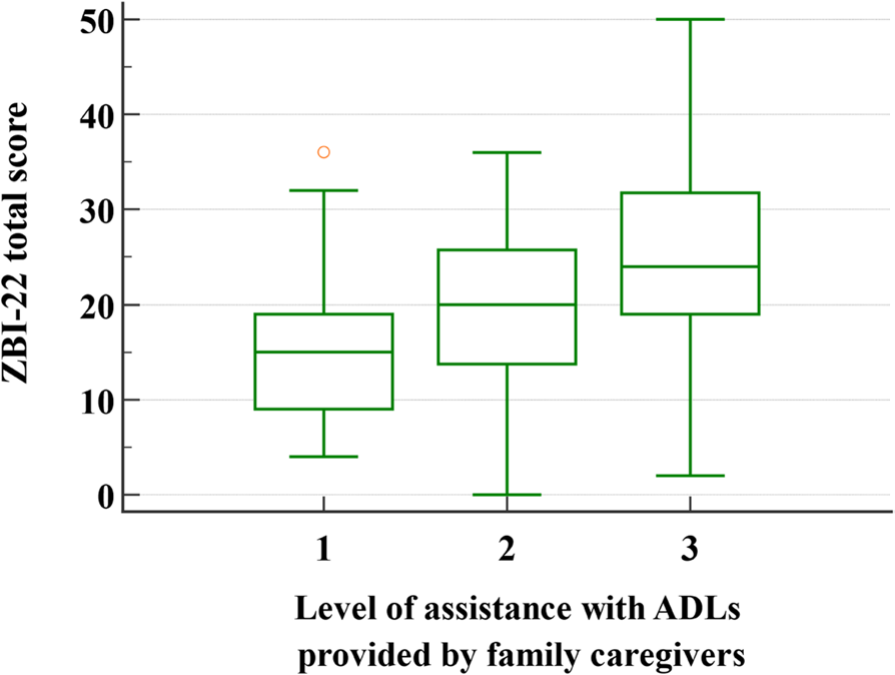
ZBI-22 score according to the level of assistance with basic ADLs provided by family caregivers

The ZBI-22 total score according to the level of assistance with ADLs provided by FCs is shown in Fig. 1. The mean ZBI-22 score for level 1 and level 2 was below the cutoff point of significant CB (mean [SD] = 15.5 [8.7] and 19.9 [9.3], respectively), while it was above the cutoff point for level 3 assistance (mean [SD] = 25.2 [8.6]).

## Discussion

This study, conducted in two Eastern Mediterranean countries, showed that a substantial proportion of FCs of incurable cancer patients experience significant CB. Among the studied variables, the only one that predicted CB was FCs’ assistance with late-loss basic ADLs.

The high prevalence of CB among FCs of incurable cancer in this study is not surprising and agrees with the findings of other studies conducted in different settings from around the world. The prevalence of CB among FCs of cancer patients in general using the ZBI-22 cutoff point of 21 ranged widely from 37 to 82% in other studies [9, 20–25]. The 59% prevalence of CB found in this study falls in the middle of this range. These studies included FCs of cancer patients at different stages and some focused on a certain type of cancer.

The mean ZBI-22 score in this study was 23, which is largely similar to that reported in other studies that included FCs of advanced cancer patients. In a study from the United Kingdom that included 105 informal caregivers of advanced cancer patients, the mean ZBI-22 score was 23 [26]. Similarly, the mean ZBI-22 score was 23 in a study from a palliative care unit in Malaysia that included 249 informal caregivers of patients with life-limiting illnesses of whom 74% had malignancy [27]. In a Thai study, the mean ZBI-22 score of 19 among 150 informal caregivers of elderly advanced cancer patients [25]. These findings suggest that there are common factors contributing to CB among FCs of patients with advanced cancer in different cultures and socioeconomic statuses.

Previous studies identified factors that may determine the presence and severity of CB; such as the absence of a helper, longer stay in the hospital, marital status, education of caregivers, type of treatment facility and the time elapsed since cancer diagnosis [9–11]. In the current study, the only variable that associated independently with significant CB was the assistance with late-loss ADLs. Although assistance with ADLs is perceived as a contributor to CB, the evidence that support this perception is not consistent. Goldstein et al. included in their study caregivers of patients with terminal cancer and found no significant association between the number of ADLs the caregivers assist with and higher CB [28]. Another Thai study found no independent association between assistance with ADLs and higher CB [25]. In our study, we examined the association between the level of assistance with basic ADLs, rather than the number of ADLs or assistance in general. We found that it is the assistance with late-loss ADLs is the one that contribute to CB. This is supported by the results of the recent study conducted by Schwartz et al. [5]. They found that only the assistance with feeding and toileting are independently associated with higher CB among caregivers of patients with cancer of different stages.

Although it may be seen by some as unnecessary, we detailed in the results section the response of FCs to the 22 items of the ZBI-22. This may serve as a guide for future research/interventions to minimize the CB of FCs in our region. Only 5 items out of the 22 scored in average > 2 on the 0–4 scale. One of these 5 items is the financial concerns. In a study from another country in the EMRO region (Iran), no enough income for living expenses was significantly associated with CB [14]. This may be a target for interventions dealing with CB. Another example is the fear of FCs of what the future holds for their related patient, which scored in average 2.4 on the 0–4 scale. Meeting the information needs of FCs and supporting them psychosocially may ameliorate their fears and consequently the burden they suffer.

A limitation of the study is that we did not study all factors that had been found to contribute to CB in other studies like the social network index of caregivers and their activity restriction [28]. Another limitation is the purposive-convenience sampling method used.

In conclusion, FCs of patients with incurable cancer experience significant CB in our region. Routine screening for CB is warranted among FCs of incurable cancer patients who assist their related patients with the basic ADLs, especially the late-loss ones. There is a need for further research to explore the needs of FCs of incurable cancer patients in the EMRO region and how to address these needs.

## Data Availability

The datasets used and/or analysed during the current study are available from the corresponding author on reasonable request.

## Abbreviations

ADLs: Activities of daily living
CB: Caregiver burden
ECOG: Eastern Cooperative Oncology Group
FCs: Family caregivers
IQR: Inter-quartile range
OR: Odds ratio
SD: Standard deviation
ZBI-22: The 22-item Zarit Burden Interview
